# Re: Lift capabilities of hyaluronic acid fillers by Marcos Borrell, Dustin B. Leslie & Ahmet Tezel (J Cosmet Laser Ther. 2011;13:21–27)

**DOI:** 10.3109/14764172.2011.586425

**Published:** 2011-05-24

**Authors:** Katarina Edsman, Anne Helander Kenne

**Affiliations:** Q-Med AB, Seminariegatan 21, SE-752 28 Uppsala, Sweden

**Sirs,**

In January 2011, a paper was published in the *Journal of Cosmetic and Laser Therapy* entitled ‘Lift capabilities of hyaluronic acid fillers’ by Marcos Borrell, Dustin B. Leslie and Ahmet Tezel (2011; 13:21–27). We have some comments about the results presented in that paper as well as some concerns about the methods and the scientific approach used.

The authors state that the lift capability of a filler depends on the gel hardness (G') and the cohesivity. They compare two different fillers: one manufactured by Allergan Inc., employer of the authors, and one filler from Medicis Aesthetics Inc., manufactured by Q-Med AB. In the paper, data are presented from measurements on the two fillers and a comparison is made regarding the hardness and the cohesivity. The paper also describes the manufacturing processes of the fillers and speculates on properties that could be commented on. However, we have focused on the scientific approach and the experimental data presented in the paper.

We do agree that the gel strength (or ‘gel hardness’, the term used in the paper) may be an important factor for the lifting capacity and that the gel strength could be measured by rheology. In fact, we presented and discussed this at the Anti-aging Medicine World Congress in 2010 (1). Rheology is usually perceived as difficult and therefore would a correct terminology have helped the readers to understand the presented data. Using the two invented terms ‘linear viscosity’ and ‘gel hardness’ for the scientific established term ‘elastic modulus’ will only confuse the readers, making it more difficult to compare the results with earlier published data (2–5). The term ‘linear viscosity’ does exist; it is sometimes used synonymously with ‘zero shear viscosity’, which is measured by rotational viscometry. The linear viscosity or the zero shear viscosity may also be calculated from a frequency sweep if the Cox–Mertz rule (6) applies to the material, but it cannot be calculated from a strain sweep measurement at 5 Hz as the authors claim.

Rheology is a useful tool for characterization of gel properties and several different rheological methods can be used to gain information about the material. The only rheological results presented in the paper are from a strain sweep measurement at 5 Hz (see their Figure 5), where the plateaus at low strains give the elastic modulus of the fillers and a shorter plateau (a plateau ending at a lower strain) is usually found for stronger gels. Normally, a strain sweep is made to determine the linear vis-coelastic region (LVR), which is used to find the settings for the subsequent measurements. A frequency sweep, as used in the reference cited in the Methods section (4), is a rather common method that could have been used but with a different experimental set-up than used in reference 4 (7). The only information gained from the strain sweep shown in the paper is the elastic modulus at 5 Hz, which could have been given in a table or in the text. Presenting the curves (Figure 5) does not give any more information; probably it is only more confusing for those not engaged in rheology measurements. The authors do not help the readers to understand the figure; rather they confuse the readers further by stating that their product “provides lift because it shows less susceptibility to yield to a given strain”. The interpretation of the rheology section in the paper is that the authors have a limited knowledge in the field.

Whether the cohesivity may or may not contribute to the lifting capacity of a filler can be discussed, but it is a fact that the cohesivity is not easy to measure, which the authors also state. Measuring the cohesivity by a compression or a tensile test is probably a good approach. Since the method used for cohesion measurements is not a standard method, and the instrumentation used is rather unusual for compression tests, the experimental set-up should have been described in such detail that the experiment could be repeated elsewhere. The actual volume of filler used should have been stated, as well as how it was loaded onto the measuring geometry, as the results most certainly are strongly affected by the size and shape of the loaded sample. It would also have been appropriate to present repeated measurements to show the reproducibility of the measurements. Another objection could be made regarding the rationale to measure the cohesivity of the product, since it is the cohesivity in vivo that would affect the lifting capacity. The authors state that the cohesivity of the filler is affected by the presence of uncross-linked/free hyaluronic acid (HA); more free HA decrease the cohesivity of the product. After injecting a filler into the tissue, the free HA will diffuse out of the gel, reducing the amount of free HA and hence increasing the cohesivity. Comparing cohesivity in the presence of free HA, as made in the paper, does not relate to the in vivo situation. In vivo, the cohesivity of the products could be completely different, even the ranking could be changed since the products may contain different amounts of free HA (8).

The other method used to qualitatively show differences in cohesion is also poorly described. It is neither easy to understand the scientific base for the experiment nor how the experiment is performed. The authors name the method ‘dye diffusion test’ but we do not believe that it is a diffusion test, as the rate of diffusion of a small molecule is as fast in a gel as it is in pure water if no interactions occur (9,10). The faster spreading rate of the dye for the Medicis product probably depends on convection that can occur in solutions and in solvents but not in gels. As can be understood from the description of the experiment, a fixed quantity of the filler is added on top of an equal amount of a buffered solution and then centrifuged, whereafter the dye is added to the top. In order to understand what may happen with the gel in the experiment some inherent characteristics of polymer gels must be understood/known. The amount of water that can be absorbed by a gel before it turns into a two phase system depends on the gel strength. A stronger gel has a more limited water absorption capacity and a weaker gel is able to absorb more liquid. Since the gel strengths are different for the tested fillers they have different water absorption capacities. Let us assume that all fillers in the study are capable of absorbing the added liquid (otherwise it is a measurement of the water absorption capacity, i.e. another measure of the gel strength). If the products are not thoroughly mixed with the solvent and allowed to swell before the centrifugation, the gels are centrifuged to the bottom of the syringe in the experiment and the results will rather reflect the difference in swelling rate/water absorption rate. For a gel to be able to swell if it is situated at the bottom of the container, below the solvent, a rearrangement of the gel particles is necessary. The rate of such a rearrangement will probably be slower for a more cohesive material. If the experiment is not described in enough detail it is not possible to critically examine the conclusions drawn by the authors. If the gels were not allowed to swell before the centrifugation, the larger spreading rate of the dye for the Medicis product could be due to convection in the solvent phase above the gel as a result of a slower swelling. In that case, an equally probable interpretation of the results from the dye diffusion test is that the Medicis product needs more time for the rearrangement of particles, and the conclusion would be that the Medicis product is a more cohesive gel.

Except for the criticism above there are many other comments that could be made: for example, there are data presented in the text as well as in their Table II without a description of the methods used; the illustration of high and low cohesion is made with different distances between the probes in their Figure 3A and B.

We appreciate the attempt the authors made to find properties that may correlate to the in vivo performance of dermal fillers, but it is also sad/disappointing to see that the scientific level of the study is so low.

We encourage the journal to publish this letter so that the publication by Borrell, Leslie and Tezel can be critically examined.
